# New insight in molecular detection of *Mycobacterium tuberculosis*

**DOI:** 10.1186/s13568-024-01730-3

**Published:** 2024-06-21

**Authors:** Seyyed Mohammad Amin Mousavi-Sagharchi, Elina Afrazeh, Seyyedeh Fatemeh Seyyedian-Nikjeh, Maryam Meskini, Delaram Doroud, Seyed Davar Siadat

**Affiliations:** 1 Department of Microbiology, College of Basic Sciences, Shahr-e-Qods Branch, Islamic Azad University, Tehran, Iran; 2 Department of Marine Biology, Faculty of Marine Science, Khorramshahr University of Marine Science and Technology, Khorramshahr, Iran; 3 Department of Mycobacteriology and Pulmonary Research, Pasteur Institute of Iran, Tehran, Iran; 4 Microbiology Research Center (MRC), Pasteur Institute of Iran, Tehran, Iran; 5 Department of Genetics, Faculty of Natural and Agricultural Sciences, University of the Free State, Bloemfontein, 9301 South Africa; 6 Student Research Committee, Pasteur Institute of Iran, Tehran, Iran; 7 Department of Immunotherapy and Leishmania Vaccine Research, Pasteur Institute of Iran, Tehran, Iran

**Keywords:** *Mycobacterium tuberculosis*, Tuberculosis, MTB, TB, Molecular diagnosis, PCR, SNPs, Molecular assays

## Abstract

*Mycobacterium tuberculosis*, the causative agent of tuberculosis, is a pathogenic bacterium that has claimed millions of lives since the Middle Ages. According to the World Health Organization’s report, tuberculosis ranks among the ten deadliest diseases worldwide. The presence of an extensive array of genes and diverse proteins within the cellular structure of this bacterium has provided us with a potent tool for diagnosis. While the culture method remains the gold standard for tuberculosis diagnosis, it is possible that molecular diagnostic methods, emphasis on the identification of mutation genes (e.g., *rpoB* and *gyrA*) and single nucleotide polymorphisms, could offer a safe and reliable alternative. Over the past few decades, as our understanding of molecular genetics has expanded, methods have been developed based on gene expansion and detection. These methods typically commence with DNA amplification through nucleic acid targeted techniques such as polymerase chain reaction. Various molecular compounds and diverse approaches have been employed in molecular assays. In this review, we endeavor to provide an overview of molecular assays for the diagnosis of tuberculosis with their properties (utilization, challenges, and functions). The ultimate goal is to explore the potential of replacing traditional bacterial methods with these advanced molecular diagnostic techniques.

## Introduction

Tuberculosis (TB), caused predominantly by *Mycobacterium tuberculosis* (MTB), remains a formidable global health challenge. Despite significant progress in its control and management over the years, TB continues to exact heavy damage to human society, with an estimated 10 million cases and 1.6 million deaths in 2022 (Meskini et al. 2023; Nour Neamatollahi et al. 2023). The World Health Organization (WHO) has identified TB as the leading cause of death from a single infectious agent worldwide, surpassing even the human immunodeficiency virus (HIV) (Kasavandi et al. 2023). MTB is the most prevalent infection and one of thirteen causes of mortality due to infections in the world (Meskini et al. 2020, 2021; Tilwani et al. 2023). These alarming statistics underscore the pressing need for innovative diagnostic tools and approaches to enhance TB detection, treatment, and control.

One of the defining features of MTB is its remarkable genetic diversity, which has significant implications for disease presentation, transmission dynamics, and drug susceptibility (Rahman et al. 2022). Molecular assays have played a pivotal role in characterizing this diversity, allowing the categorization of MTB strains into various lineages and families. This information is invaluable for epidemiological studies, as it helps trace the spread of TB outbreaks and informs the development of region-specific control strategies (Rezaei et al. 2023; Yin et al. 2023). Furthermore, the emergence of drug-resistant MTB strains poses a formidable threat to global TB control efforts. Genotypic assays have revolutionized the detection of drug resistance mutations, offering rapid and accurate insights into the susceptibility profile of clinical isolates (Kavya et al. 2023). These assays such as line probe assay (LPA) and whole-genome sequencing (WGS), have transformed clinical decision-making by enabling tailored drug regimens and reducing the risk of treatment failure (Van Rie 2023).

Traditional diagnostic methods for TB, such as sputum smear microscopy, light emitting diode fluorescent microscopy (LED-FM), and culture-based techniques, while reliable to some extent, are time-consuming, labor-intensive, and often suffer from low sensitivity, particularly in cases involving extrapulmonary TB or drug-resistant strains (Jakhar et al. 2020; Reza et al. 2013). Despite the gold standard of MTB diagnosis being cultivation on the Löwenstein-Jensen (LJ) medium, technicians will have to deal with contamination risks. Detection of MTB through microscopic techniques (simple smear acid-fast staining and LED-FM) can be challenging due to its small size (Zaporojan et al. 2024). On the other hand, patients feel exhausted by spot-spot-morning (SSM) and spot-morning-spot (SMS) sampling methods. The advent of molecular biology and biotechnology in the latter fifty years of the 20th century heralded a new era in TB research. In the quest for more accurate, rapid, and efficient TB diagnostics, molecular assays have emerged as promising tools that have revolutionized our ability to detect and characterize MTB with unprecedented precision (Forero and Chand 2023; Nour-Neamatollahi et al. 2018). Molecular assays, driven by nucleic acid amplification techniques (NAATs) such as polymerase chain reaction (PCR), loop-mediated isothermal amplification (LAMP), and DNA sequencing, have facilitated unprecedented insights into MTB’s biology (Lee et al. 2019). These assays have enabled the elucidation of the bacterial’s genome, revealing a trove of genetic information pertinent to its virulence, drug resistance, and evolution (Ghosh et al. 2022; Prajwal et al. 2023).

The genomic mapping of MTB is explicitly outlined in various databases, including tools provided by the National Center for Biotechnology Information (NCBI) and various published papers (Sayers et al. 2022). These resources enable us to establish a reliable framework for developing molecular methods (Lorente-Martínez et al. 2022). The identification and characterization of resistant genes are pivotal for prescribing drugs in the patient’s treatment process (Alexander et al. 2022). Once various molecular assays are designed for detecting MTB and related drug resistance to facilitate effective therapy, a concise review becomes essential. This review provides experts with both general and specialized information on the molecular assays employed in TB diagnosis. The current review compiles and synthesizes data extracted from diverse papers delineating molecular assays along with their properties, including utilization, challenges, and mechanisms of function.

As we embark on the next frontier of TB research, the integration of omics technologies, artificial intelligence (AI), and advanced imaging promises to further enhance our understanding of MTB. Challenges such as the emergence of drug-resistant strains, the persistence of latent infection, and the need for point-of-care diagnostics remain formidable obstacles. This review will explore the evolving landscape of TB research and the prospects for innovative solutions. Besides, delves into the world of molecular assays targeting MTB, providing an in-depth exploration of their principles, applications, advantages, and limitations. By elucidating the evolution and current state of these assays, we aim to offer a comprehensive perspective on their role in TB diagnosis and management. Furthermore, we will highlight recent advancements and ongoing research efforts in this field, underscoring their potential to shape the future of TB diagnostics.

## Epidemiology and limitations in low-income countries of the Middle East and South Asia

While molecular assays prove to be effective and straightforward, they pose a significant drawback due to their high cost (Salvador et al. 2022). This issue becomes more pronounced in the Middle East and South Asia where impoverished countries such as Afghanistan, Pakistan, Iraq, Syria, Yemen, and Gaza are situated in 2021 and 2022 (Mate et al. 2017; Nour Neamatollahi et al. 2023). The lack of accessible molecular rapid tests for the early diagnosis of TB has led to an increased prevalence in low-income countries, particularly in conflict-ridden countries like Yemen (Basamed and Alamoudi 2023).

The WHO’s announcement in 2021 revealed alarming statistics regarding the prevalence of TB in the Middle East, especially in Pakistan. According to the report, 8% of the total TB cases in the Middle East are attributed to the region, with 71% of these cases specifically linked to Pakistan. Furthermore, there has been an 8% increase in multi-drug-resistant TB (MDR-TB) in the Middle East, accounting for about 8% of all MDR-TB cases globally (WHO 2021a). The TB caused by MDR-TB strains has further exacerbated financial burdens due to the complexities associated with treatment (Asres et al. 2018; Madadi-Goli et al. 2024; Meskini et al. 2023).

## Genome sequence and mutations

Man has long strived to combat the scourge of TB, a battle spanning 9000 years. Presently, our goal is to eradicate this disease from the face of the Earth. In pursuit of this noble objective, the WHO has recently endorsed gene identification and sequencing-based diagnostic methods as the most potent tools for disease detection and management ((WHO), 2018). The arsenal of technologies for bacterial genome sequencing and identification comprises two key advancements: WGS, introduced in 1998, and next-generation sequencing (NGS), introduced in 2009 (Barba et al. 2014; Wang et al. 2022). These groundbreaking technologies excel in identifying both species and mutations within the 4.4 million bp of the *Mycobacterium* genome, which encompasses a staggering 4000 genes (Advani et al. 2019; Hadifar et al. 2022).

**Fig. 1:**
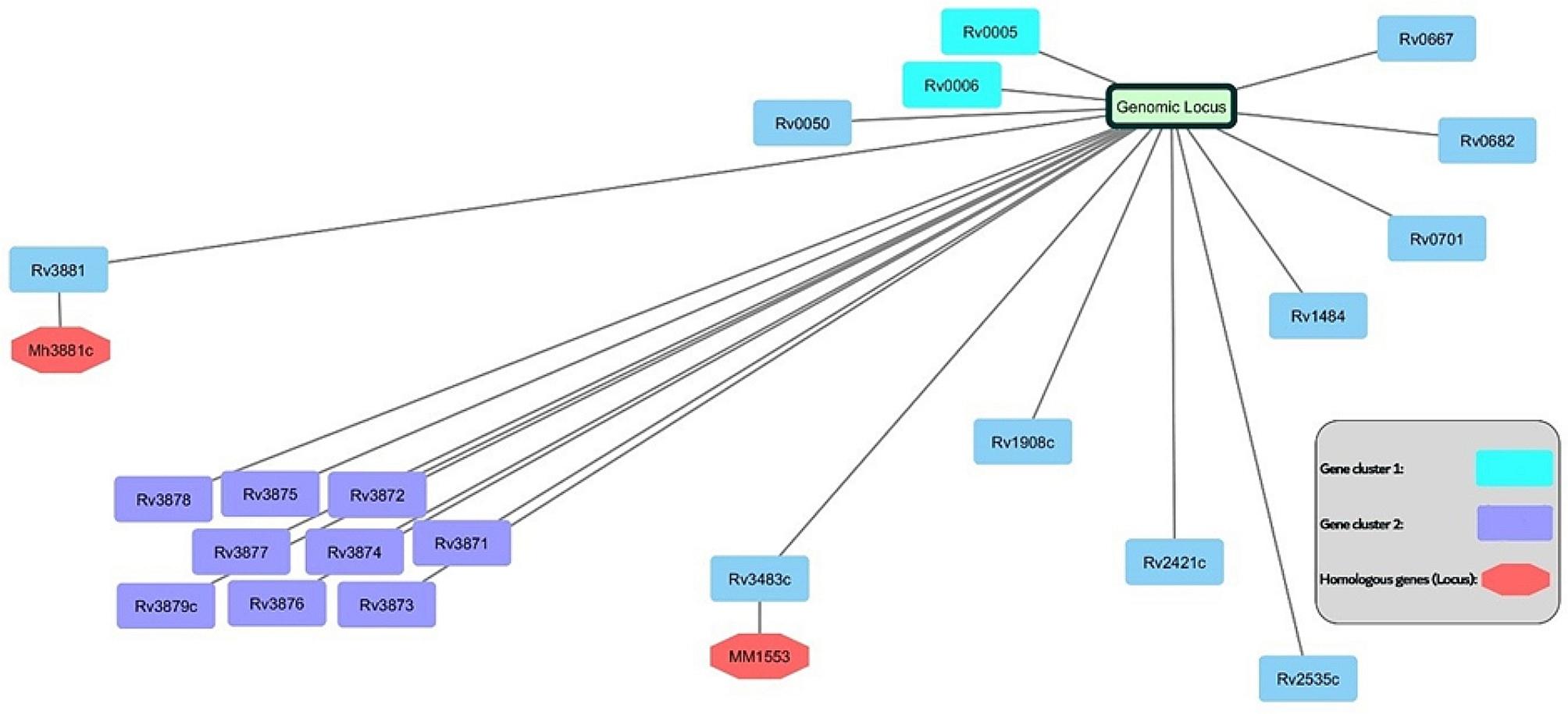
Genomic locus and cluster details of MTB. Genes responsible for expression of the virulence factors alongside other genes are generally located in genomic clusters. Gene clusters are responsible for antibiotic resistance and virulence features of bacteria. There are many clusters in the bacterial chromosome, and this figure shows a scheme of two gene clusters and several genes with their homologous genes

*Mycobacterium*’s virulence genes are clustered within approximately 50 kb of its chromosome. Upon closer examination of the *Mycobacterium* genome, a virulence cluster becomes apparent, specifically located in the region between Rv3871 and Rv3879c, known as RD-1. Remarkably, this cluster aligns perfectly with the open reading frame (ORF) (Ganguly et al. 2008) (Fig. 1). Homologous gene clusters also play a pivotal role in elucidating this bacterium’s virulence, including *MM1553* (homologous to Rv3483c) and *Mh3881c* (identical to Rv3881c) (McLaughlin et al. 2007).

Mycobacteria are among the bacterial genera that exhibit a high degree of genetic mutations (Coscolla and Gagneux 2014). According to the WHO, this bacterium (MTB) has accumulated a staggering 17,000 mutations until 2021 (WHO 2021b). These mutations play a pivotal role in bolstering bacterial survival by conferring resistance to antibiotics (Akrami et al. 2023). The identification of multilocus sequence (*hsp65, rpoB*, and 16S ribosomal DNA (rDNA) gene) assumes paramount importance in optimizing antibiotic therapies for effective treatment (Tarashi et al. 2023). A plethora of diagnostic methods is designed around genome analysis and the investigation of antibiotic resistance (Georghiou et al. 2023; Su et al. 2019). Mutations in genes such as *rrl*, *rpoB*, *gyrA*, and *gyrB* are responsible for instilling resistance to linezolid (LZD), rifampicin (RIF), and levofloxacin (Akrami et al. 2023; Chien et al. 2016). Additionally, other genes such as *pepQ*, *crfA*, *Rrs*, and *rrs* can induce resistance against clofazimine, imipenem, amikacin (AMK), and streptomycin (STR) (Sreevatsan et al. 1996; Wang et al. 2022). These genetic changes predominantly manifest as alterations in base pairs or nucleotides. Genotypic changes, in turn, bring about phenotypic modifications in microorganisms, thereby influencing the production of virulence proteins (Martinez and Baquero 2000). Subsequently, these changes in phenotype trigger the activation of resistance mechanisms (Saghi et al. 2015; Woodford and Ellington 2007).

RIF binds to the RNA polymerase β subunit, that is encoded by the *rpoB* gene, and inhibits protein transcription (Rossau et al. 1997). In over 95% of RIF-resistant strains, mutations are observed within an 81-base pair region (codons 507–533) of the *rpoB* gene (Zaw et al. 2018). Automated DNA sequencing has identified more than 50 mutations in this region, with most involving point mutations in codons 516, 526, or 531 (Morgan et al. 2005). Research indicates that over 90% of RIF-resistant TB is also resistant to isoniazid (INH), making RIF resistance an effective strong marker for MDR-TB (Bahraminia et al. 2021; Mousavi Sagharchi and Mahmoudi Nasab 2023).

## Molecular diagnosis, specified and non-specified

Molecular assays were developed after DNA structure was discovered. The specificity and sensitivity of molecular methods have led to improved molecular assays for detecting disease agents. Several of them are general and can be applied to all species and taxa. In contrast, others are specific to each microorganism or organism gene (s). Methods based on molecular biology, such as real-time PCR (RT-PCR), microarrays, PURE-LAMP, NGS, and WGS, can detect genes in all taxa. A variety of molecular assays, tests, methods, and kits, including those for Amplicor MTB, Cobas TaqMan MTB, E-MTD, FluoroType MTB, LPA, Anyplex MTB, Xpert MTB, and Genedrive MTB, are available for accurate detection of MTB resistance genes. The newest detection method is TB-CRISPR, a CRISPR-based approach.

## Assays and methods

### RT-PCR

Through PCR, DNA replication can be simulated in vitro, allowing DNA amplification. Different molecular methods can be used to detect amplified DNA after the replication process. As part of the PCR system, a florescent probe is attached to the free R at the 5’ terminal for the same-time detection of DNA (Chen et al. 2022). The RT-PCR, or quantitative PCR, is capable of detecting amplified genes and luci in MTB, including IS*6110*, IS*1081*, 16S *rRNA*, 23S *rRNA*, *hupB*, *gyrA*, *mpt64*, and other virulence genes (Sánchez-Carvajal et al. 2021).

**Fig. 2:**
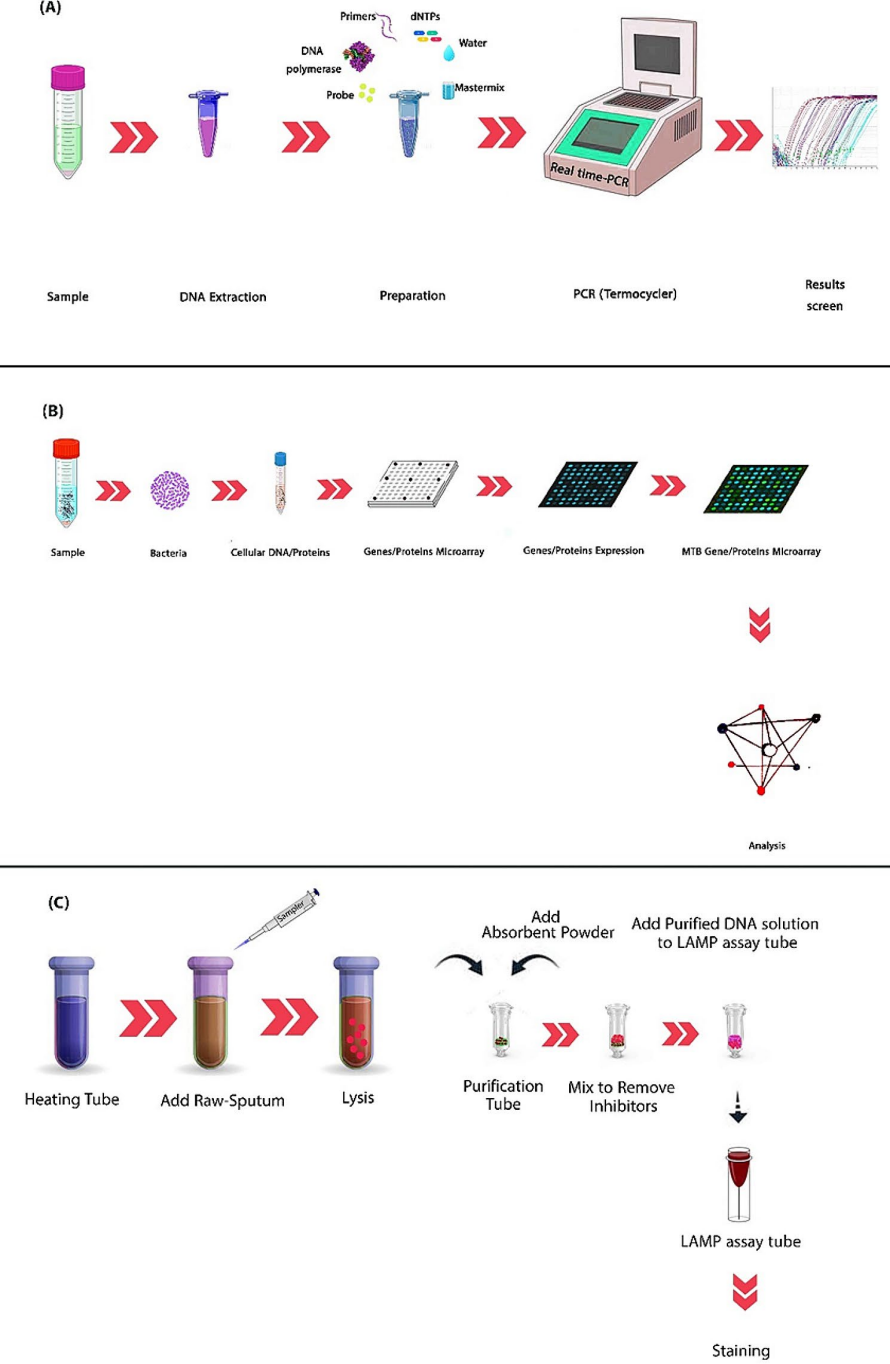
Quick review of RT-PCR, Microarray, and PURE-LAMP. (**A**) RT-PCR: The DNA sample should be extracted by enzymic or similar techniques; primers, DNA templates, Taq DNA polymerase, dNTPs, DDW, and Mg ^2+^ or Mn^2^^+^ ions should added before PCR. Next, the DNA is amplified by PCR, and any DNA present in the sample will be identified by probes in fluorescent light. **(B) **DNA microarray: MTB proteins, genes, or SNPs are prepared separately and stored in a suitable sterile container. In the next step, fluorescein is used and they are labeled. To call MTB factors in laboratory conditions, proteins are expressed and purified. After centrifugation, the samples are washed with buffer. Subsequently, the sample is placed on the microarray platform and the result is recorded by the microarray scanner. All the steps of hybridization, scanning, result interpretation, washing, and drying should be done according to the manufacturer’s instruction kits which can be different. **(C) **PURE-LAMP: This method is the easiest way to detect MTB in molecular assays, that extract the DNA directly and amplify it without using PCR. MTB is detected after amplification in the direct method by turbidimeter or fluorescence staining (Vincent et al. 2023)

To amplify a sequence, a cycle contains three steps - denaturation, annealing, and extension that are based on temperature changes (Garibyan and Avashia 2013) (Fig. 2-A). Besides, we need to prepare all of the components needed for DNA replication, such as primers, DNA templates, Taq DNA polymerase, dNTPs, double distillation water (DDW), and Mg^2+^ or Mn^2+^ ions as co-enzymes. Amplified targeted genes are usually analyzed using different methods, such as gel electrophoresis (Chen et al. 2021). However, in the RT-PCR method, an on-screen monitor will display real-time expression levels of genes.

### Microarray

Microarray means the miniaturization of thousands of measurements on a single platform (Brambilla et al. 2021). Molecular analysis of nucleic acids is predominantly employed for determining the presence of microorganism-caused infections. The DNA microarray is utilized to ascertain genotypic data, particularly single nucleotide polymorphisms (SNPs), at a high density and to investigate transcription and gene expression (Behzadi and Ranjbar 2019; Brambilla et al. 2021). Microarray serve as valuable tools for specific identification and high-throughput (HT) detection of microbes, revealing the disease mechanisms, and medicinal targets (Feng et al. 2020; Pandey et al. 2021). Microarray gives us a chance to analyze of huge genomic data in one simple test (Behzadi et al. 2014).

With its high sensitivity, accuracy, and capacity, the DNA microarray assay possesses a distinct advantage in monitoring various genes at the same time compared to other diagnostic tools (Lee et al. 2010). Factors affecting the analytical signal in DNA microarray analysis include probe length and characteristics, hybridization temperature, incubation time, and buffer composition (Jaksik et al. 2015). High-quality and abundant DNA are crucial prerequisites for this method. SNP analysis is the most common application in microarray analysis (de Vries et al. 2022). Low DNA quantity Samples yield low-quality SNPs and are susceptible to genetic errors (Yagasaki et al. 2022). Depending on the test’s purpose and framework, various types of samples can be employed (Chu et al. 2023; Jin et al. 2022). In a typical DNA microarray assay, desired nucleic acid fragments are labeled, washed, and dyed with a fluorescent dye such as Cyanine3 (Cy3) and Cy5 for detection (Fig. 2-B) (Taguchi et al. 2021).

This pioneering assay can detect MTB and its drug-resistant variants in sputum samples (Zhang et al. 2012). Microarray can detect six SNPs for lineage identification and *gyr*A, *gyr*B, *rrs*, *eis*, *katG, inhA*, *rpoB, ahpC* resistance genes (Nguyen et al. 2019). Although microarray can detect many genes at the same time, for simplicity of procedure it can be used in identifying mutations in the *rpoB*, *gyrA, and pncA* genes, predicting resistance to RIF, fluoroquinolones, and PZA, respectively (Havlicek et al. 2019; Wade et al. 2004). For this purpose, samples should be placed into a microarray platform and recorded the results with a microarray scanner such as LuxScan™ from CapitalBio Co. (Sun and Sun 2021).

### Procedure for ultra-rapid extraction (PURE)

The PURE-LAMP, initially introduced by Notomi in 2000, presents a novel, uncomplicated, and contamination-resistant (Notomi et al. 2000). It employs NAA combined with ring-LAMP to MTB detection. One of its notable advantages is its rapid processing and LAMP ability to replicate and amplify huge amounts of DNA. Furthermore, the PURE-LAMP stands out for its cost-effectiveness, as it doesn’t require a thermocycler or expensive equipment (Jekloh et al. 2022; Notomi et al. 2000).

The PURE exhibits a remarkable capability for the swift and accurate dignosis of MTB (Ou et al. 2014). In fact, it can detect MTB within just two hours, a significantly shorter time frame compared to traditional culture methods. TB-LAMP is the specified LAMP for TB developed by Eiken Chemical Company in Japan (Ou et al. 2014). This test entails three key stages: sample preparation, amplification, and visual detection using ultraviolet (UV) light emitted from the test tube (Eddabra and Ait Benhassou 2018b) (Fig. 2C). All procedure is done in a tube with an isothermal reaction at 63 °C (Noor et al. 2015). The sensitivity of this test for point sputum is approximately 70.67% and based on the combination of three sputum, its specificity is 98.32% (Eddabra and Ait Benhassou 2018b; Ou et al. 2014). This method is recommended for TB diagnosis confirmation alongside other diagnostic methods, especially in cases with false-negative results (Eddabra and Ait Benhassou 2018b). It should be noted that this method is unable to identify and diagnose DR patterns (Bojang et al. 2016).

### NGS

**Fig. 3:**
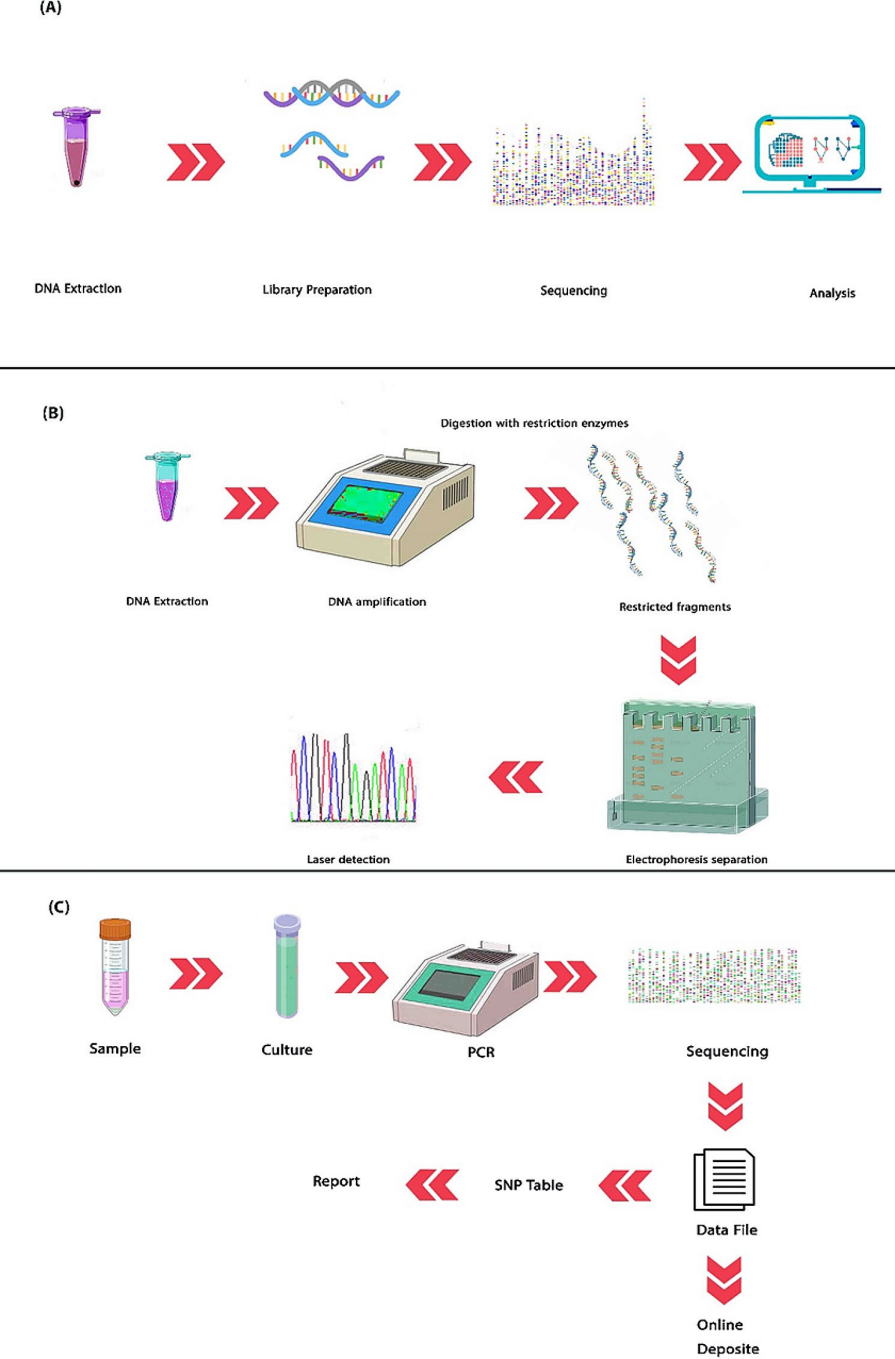
A brief review of NGS, RFLP, and WGS.** (A) **The NGS: this technology is one of the sequencing methods approved by WHO for diagnosis. After the DNA extraction and PCR performance, DNA should be sequenced, and results will be uploaded to databases or analyzed by bioinformatics tools. **(B) **RFLP analysis: this method is one of the oldest methods to detect MTB. In this method, DNA after amplification, is restricted by enzymes and labeling. next, gel electrophoresis is done for the separation of different fragments of DNA. Detection of fragments is done with laser emission. **(C) **The WGS: this technology was presented after the introduction of molecular biology to identify microorganisms based on their genes. The DNA is separated by different methods (e.g., enzymic method) and amplified by PCR. Genome sequencing has several steps: (1) DNA cutting (to identify different genes, DNA is cut into pieces), (2) to identify the cut pieces (coded bands are added to the DNA fragments), and (3) the sequencing of DNA bases (is done by sequencing method and analyze). Strain identification can be done with genetic detection

By growing the molecular sciences and emerging bioinformatics, new methods such as NGS are being developed to sequence nucleic acids. In order to perform NGS, several steps are required: (1) Fragmentation of DNA (can be implanted by various methods, including enzymatic digestion and PCR), (2) Preparation of the library (modification of DNA segments and addition of sequencing adaptors to segments for sequencing in the next step) (Hess et al. 2020), (3) Sequencing (NGS sequencer devices work based on massively parallel sequencing, library uploads into the system for sequencing, and different devices have different matrices and matrix design methods. Illumina® is one of the most popular systems), and (4) Analysis (sequences, nucleic bases, and their order, identified sequences must be analyzed by bioinformatics tools for data interpretation. This step involves finding variants and mutations) (Qin 2019) (Fig. 3A).

As a result of mutations in MTB and difficulties in identifying them, new devices and technologies are being used to identify drug-resistance genes. An NGS method is very useful for revealing mutations in MTB sequences (Beviere et al. 2023). Different species follow similar steps, but there are a few differences, such as in library preparation kits and interpretation databases. In addition to the data and methods available for MTB, there are several methods and libraries available as well: TBDReaMDB, MUBII-TB-DB, PhyResSe, TBDR, DRAGdb, Resistance Sniffer, ReSeqTB, PointFinder, and Deeplex® Myc-TB (Beviere et al. 2023; Dookie et al. 2022a). As an example, Deeplex Myc-TB (Genoscreen, Lille, France) identifies, genotypes, and performs DST for MTB and is an ultradeep sequencing method (MacLean et al. 2020).

### RFLP

One of the most widely used methods for genotyping MTB is the RFLP, a combination of a restriction enzyme and a clone-specific method (Ei et al. 2019). Since 1990, MTB IS*6110*-RFLP genotyping has been established as the standard method for MTB strain typing that is based on the presence of the IS*6110* insertion sequence, this method can be used as a biomarker.

RFLP refers to the detection of differences in homologous DNA sequences through the presence of fragments of different lengths after digestion of the DNA samples with specific restriction endonucleases. In terms of molecular markers, RFLP is specific for a combination of clones and restriction enzymes. It is common for RFLP markers to be co-dominant (both alleles are detected in a heterozygous sample) and locus-specific. After the digested DNA sample has been separated by gel electrophoresis, an RFLP probe hybridizes with one or more fragments of the digested DNA sample, revealing a unique blotting pattern characteristic of the genotype at the isolated locus (Fig. 3-B). A RFLP probe is typically composed of a short, single- or low-copy genomic DNA or cDNA clone. Genotyping, forensics, paternity tests, hereditary disease diagnostics, and forensics use RFLP probes for genome mapping and variation analysis (NCBI).

Due to the high variability in the number and position of insertions, it is a useful tool for strain differentiation (Arora et al., 2020). Also, in comparison with conventional molecular phenotypic methods, it is the most stable and reliable (Kone et al. 2020). One of the disadvantages of this method is that it is not suitable for isolates with low copy numbers (Bakhtiyariniya et al. 2022). In spite of this, semi-automated RFLP has been found to be a robust and promising method for routinely typing MTB (Said et al. 2016).

### WGS

The WGS encompasses several key steps, including the culture of isolates, DNA extraction, sequencing, bioinformatics analysis, and data interpretation (Mintzer et al. 2019) (Fig. 3-C). SNPs analysis is the most commonly employed bioinformatics approach for evaluating the similarity of isolates. In TB reference laboratories, the use of the WGS is increasing (Jajou et al. 2019). WGS is used to detect resistance and predict sensitivity and trace transmission of MTB (Takiff and Feo 2015). This technology allows the detection of mutations outside the target area of the assays (Shea et al. 2017). WGS has made it possible to distinguish between infection and recurrence, and also, does not have the limitations of normal drug susceptibility tests (DSTs) and rapid molecular tests. Sensitivity to first and second-line drugs was determined using WGS (Iketleng et al. 2018). The DNA is then split into smaller pieces to be sequenced. The results should be analyzed quickly in a clear way to enable the use of WGS data (Lee and Behr 2016).

This is more cost-effective compared to NGS (Lázaro-Guevara et al. 2023) and provides a wealth of information surpassing that of other molecular techniques (Lee and Behr 2016), so it can be utilized in mixed infections (Tarashi et al. 2017). WGS is one of the strong technologies in comparison with other genome sequencing tools such as RFLP and Mycobacterial interspersed repetitive-unit–variable-number tandem-repeat (MIRU-VNTR) (Vaziri et al. 2019). It boasts the highest discriminatory capability among established methods (Baert et al. 2021). This approach enables the assessment of low-frequency variations and the detection of single nucleotide and rare mutations (Lázaro-Guevara et al. 2023; Schwarze et al. 2018). WGS finds extensive application in the diagnosis of disease outbreaks (Rubin et al. 2022).

Sekizuka et al. have developed a total genotyping solution (TGS) for TB (TGS-TB) based on WGS. TGS-TB allows for more accurate and differentiated strain typing in clinical specimens and epidemiological research (Sekizuka et al. 2015). The results of TGS-TB align with those of conventional molecular genotyping methods through in-silico analysis (Xiao et al. 2023). This bioinformatics platform stands out as one of the most widely developed and user-friendly tools available (Macedo et al. 2018). The TGS-TB enables the detection of genetic resistance to a broad range of first- and second-line drugs (Takii et al. 2019; Van Beek et al. 2019). TGS-TB, when combined with the KvarQ algorithm, enhances efficiency by saving time and enabling batch upload of samples (Sekizuka et al. 2015). TGS can continuously read long single molecules and produce facilitative assembly. The results of the tool are conveniently displayed on one screen, streamlining the process of evaluation and data collection (Van Beek et al. 2019). MTB’s TGS lineage analyses occur due to phylogenic core and complete genome sequencing. Genome phylogenetic data are based on VNTRs single nucleotide variants (SNVs), and analysis of the IS*6110* insertion site (Iwamoto et al. 2019; Panossian et al. 2018).

### Truenat

The Truenat endorsed by WHO in 2020 for the detection of microorganisms in suspected samples (Ngangue et al. 2022). Truenat drew attention to itself in Coronavirus disease 2019 (COVID-19) (Premraj et al. 2020). Truenat utilizes the chip-based RT-PCR to detect pathogens such as MTB from DNA (Meaza et al. 2021). Truenat [MTB, MTB RIF, and MTB Plus (Molbio Diagnostics, Goa, India)] are applicable to detect RIF-resistance strains. As we mentioned, different kits are available for MTB DNA extraction; to utilize the Truenat we should apply the Trueprep for DNA extraction. Truenat contains some steps to get results: (1) DNA extraction by Trueprep: liquefaction of sputum, adding lysis buffer, incubation, adding binding reagent, washing by wash buffer, (2) RT-PCR: extracted DNA should be placed on chip in the Truenat device, on the other hand, extracted DNA should be amplified by PCR on ABI 7500, and (3) Results should be compared with each other with the same master mix (Nikam et al. 2013; Premraj et al. 2020).

### Cobas TaqMan MTB

The Cobas TaqMan MTB stands as one of the most extensively employed molecular detectors for detecting MTB that utilizes NAA (Park et al. 2017). Cobas TaqMan MTB relies on RT-PCR (Causse et al. 2011). To detect the DNA of MTB, the Cobas TaqMan MTB employs the TaqMan MTB probe, amplifying a segment of the 16S rRNA gene (Bloemberg et al. 2013). Executed in two sequential steps, the COBAS TaqMan MTB encompasses sample DNA preparation and RT-PCR (Diagnostics 2009; Lee et al. 2013). This procedure begins with sample sterilization, followed by centrifugation at 60 °C for 45 min and lysis. This method is primarily designed for liquid, decontaminated, and concentrated specimens from respiratory-TB patients, so it applies to non-respiratory and other clinical specimens. Modern microbiology laboratories commonly employ this method for TB diagnosis. It’s essential to note that this method is costlier than the traditional culture method, yet it significantly contributes to TB diagnosis (Causse et al. 2011).

### Gen-probe enhanced MTB Direct (E-MTD)

E-MTD represents a modified iteration of Gen-Probe MTD (E-MTD; Gen-Probe, Inc., San Diego, California). It boasts a shortened amplification time and accommodates larger sample volumes while dispensing with both hybridization control and amplification termination steps (Bergmann et al. 1999). Notably, this test received its initial approval from the Food and Drug Administration (FDA) in 1995 (Smith et al. 1999). The execution of this method calls for the utilization of both a thermal block and a light meter. DNA serves as the primary component in the E-MTD test (Bergmann et al. 1999; Gangania et al. 2017), which has been formulated through the transcription amplification system, an NAA approach (Yu et al. 2022). It is important to acknowledge that E-MTD does possess certain limitations, including the potential for false positive or negative outcomes. Therefore, the interpretation of test results should always be grounded in the broader clinical context (Bergmann et al. 1999).

The E-MTB is a direct test for detecting MTB in respiratory samples. Typically, this test can be completed in an average of three and a half hours (Smith et al. 1999; Wu et al. 2019). For negative smear samples, if the initial test yields a negative result, a second sample should undergo testing. Should the second sample also return a negative result, it is advisable to inform the healthcare provider to consider investigating the presence of inhibitory substances (Pagaduan and Altawallbeh 2023). A positive E-MTD test result is sufficient for diagnosing TB. This laboratory procedure is commonly employed when there is a strong suspicion of TB (Gangania et al. 2017). Overall, this method is dependable, expeditious, and straightforward, effectively reducing the risk of contamination during testing (Smith et al. 1999).

### LPA MTB

**Fig. 4:**
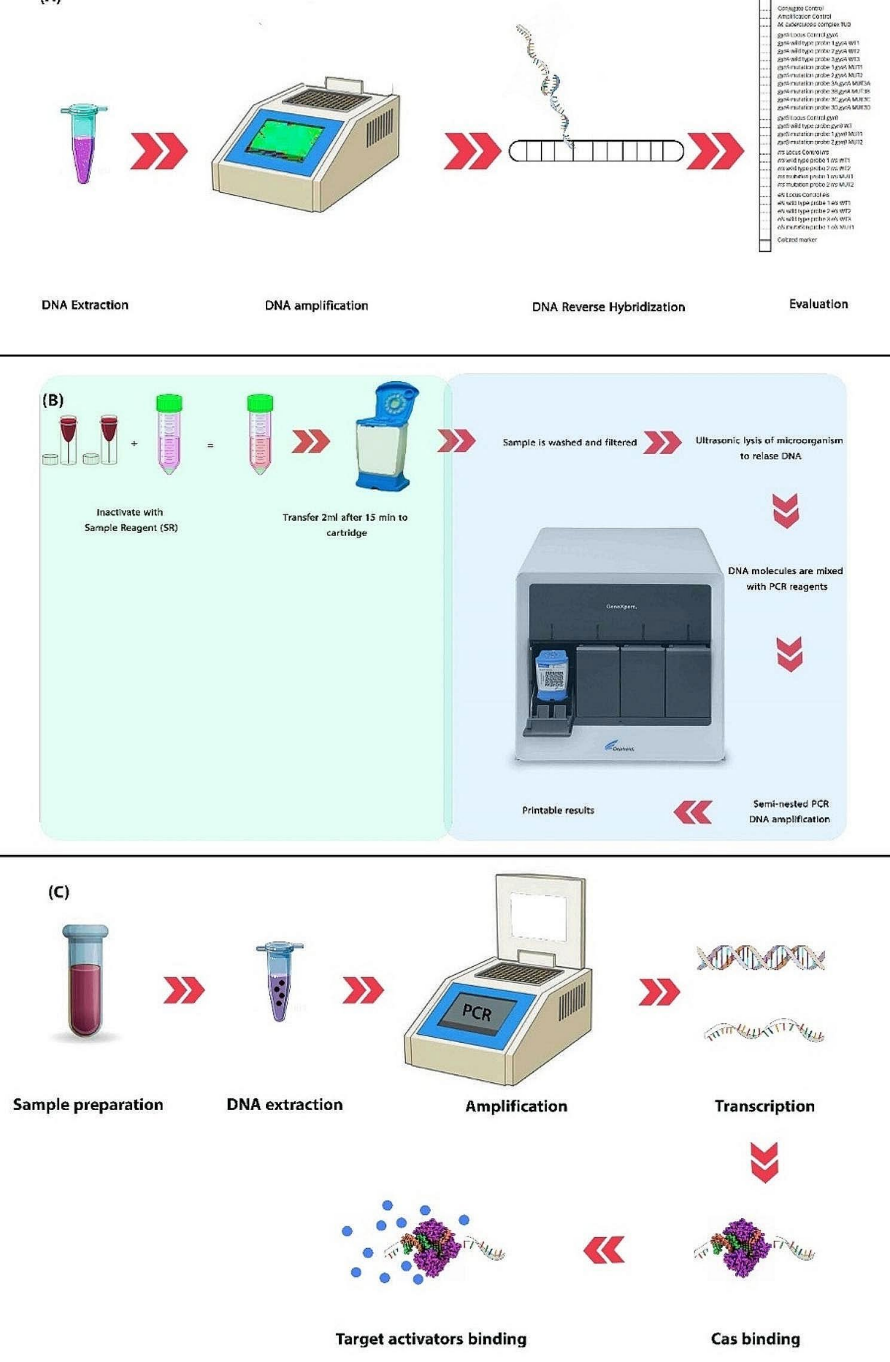
Quick view on LPA, Xpert, and TB-CRISPR.** (A) **LPA: This is a molecular line probe assay. It contains specified probes for MTBC and also probes for common RIF and INH mutations (Dorman et al. 2012). The expectorated sputum samples were decontaminated, then the pellet was suspended in a phosphate buffer. Then DNA was extracted, and amplified by PCR. Amplified nucleic acids RH to specific DNA probes bound on strips. In the final step, the DNA strip is evaluated by color formation (Dorman et al. 2012). **(B) **The Xpert MTB/RIF assay: sample preparation and reagent adding, transferring to the related cartridge (green section), and putting into the device for analysis and screening the results (blue section). **(C) **TB-CRISPR: this is a new method presented by different scientists with different protocols, this method uses the Cas protein to identify MTB in samples. After DNA extraction and amplification, transcription of T7, Cas protein will bind to the occurred complex, and target activators will bind to the complex to detect MTB’s DNA.

LPA utilizes a DNA strip technology with a cellulose acetate membrane strip. LPA relies on reverse hybridization (RH) of amplicons to immobilize membrane-bound probes, enabling the detection of mutations at frequently mutated codons (509 to 534) by using multiple probes (Mäkinen et al. 2006; Nguyen et al. 2019). In brief, the LPA process consists of three stages: DNA extraction, PCR, and RH. These three stages are conducted in separate rooms with limited access and a one-way workflow (Yadav et al. 2013). LPA can be performed using DNA extracted from clinical specimens or cultured samples. PCR is used to amplify the resistance determinant region of the related genes, followed by biotinylation of the PCR products and hybridization by probes and immobilized on a strip (Nguyen et al. 2019) (Fig. 4A). LPA is designed in three modules: module 1 identifies INH and RIF resistance by targeting the *rpoB, katG*, and *inhA*. Module 2 detects aminoglycoside resistance by targeting *rrs* and *rpsL*. Module 3 can detect mutations in *gyrA* and *embB* (Molina-Moya et al. 2015). the LPA exhibits high specificity, and high sensitivity in the early detection of MDR-TB, making it a valuable tool for the early identification of DR-TB, particularly in countries with a high TB burden (Yadav et al. 2013). LPA-based methods such as GenoType MTBC (Hain Lifesciences, Germany), and INNO-LiPA Mycobacteria (Inno-genetics, Belgium) are available for accurate diagnosis of TB and other mycobacterial diseases (Noor et al. 2015).

The GenoType® *Mycobacterium tuberculosis* plus (MTBDRplus) and GenoType MTBDRsl (Hain Lifescience, Henren, Germany) are direct detection methods designed to identify the presence of MTBC and derived from LPA (Somoskovi et al. 2008). These methodsconcurrently identify mutations within the *rpoB*, *katG*, and *inhA* genes (Bwanga et al. 2009). Common mutations in the *rpoB*, *katG*, and *inhA* genes are responsible for resistance to the primary anti-TB drugs, RIF, and INH (Barnard et al. 2008). In more than 95% of cases, TB resistance to RIF is linked to mutations occurring within an 81-base pair segment of the *rpoB* gene (Somoskovi et al. 2006). Resistance to INH is a more intricate process involving several genes, including *katG* and the *inhA* promoter region (Barnard et al. 2008; Seid et al. 2022; Takawira et al. 2017; Zaw et al. 2018). The procedure for this method can be summarized as follows: DNA extraction, PCR, RH, and the detection of mutations (Zaw et al. 2018).

Additionally, the GenoType® MTBDRplus can identify additional mutations in the *rpoB* gene, and the promoter of the *inhA* gene (Somoskovi et al. 2006). In this, resistance to INH is recognized through probes targeting the *katG* and *inhA* genes (Zaw et al. 2018). Different levels of resistance to INH can be attributed to mutations in the *katG* gene and the *inhA* promoter region, resulting in higher and lower frequencies of resistance, respectively (Kohli et al. 2021). The primary advantage of this method lies in its automated detection step, which allows for the parallel processing of up to 48 samples. As a result, this method can be carried out with a high degree of accuracy, significantly reduced reporting times, and enhanced throughput, making it suitable for high-volume laboratories even without prior experience in routine molecular assays. However, it’s important to note that this method may not a 100% detection for INH and RIF resistance (Somoskovi et al. 2008).

### Xpert MTB/RIF

WHO-recommended diagnostic tool called Xpert MTB/RIF (Xpert) offers rapid and automated NAA, enabling the simultaneous detection of RIF resistance (Kohli et al. 2021). Notably, the FDA has approved Xpert for use with raw sputum specimens and concentrated sputum sediment only. Xpert is widely employed for the concurrent identification of MTBC and RIF resistance in sputum samples. The assessment for RIF resistance is integral, whether the patient is at risk or not, and it cannot be separated from the TB diagnosis (Wu et al. 2020; Zong et al. 2019). This versatile Xpert can be implemented across all levels of the healthcare system. Detailed guidelines and extensive practical information regarding the implementation of this method have been issued by WHO (Organization 2014). The Xpert is utilized for the detection of MTBC based on DNA; represents one of the automated diagnostic assays (Sharma et al. 2015). This particular method possesses the capability to concurrently identify the MTBC and mutations linked to RIF resistance within the *rpoB* gene (Banada et al. 2010; Bunsow et al. 2014; Kohli et al. 2021). Outcomes are obtainable with minimal technical delay, typically within a two-hour timeframe from test initiation. What sets the Xpert apart is its integration of DNA extraction, nested PCR for amplification, and labeling with fluorescent dye. Process and detection occurs into a self-contained testing unit called the GeneXpert cartridge (Blakemore et al. 2011). All post-sample loading steps are executed as fully automated, autonomous measurements (Nicol et al. 2013) (Fig. 4-B). Moreover, the assay sample reagent used for sputum liquefaction possesses potent anti-TB properties, significantly mitigating biosafety concerns during the procedure (Banada et al. 2010; Kohli et al. 2021). Importantly, Xpert demonstrates the capability to detect both viable and non-viable bacteria (Miotto et al. 2012).

By hemi-nested PCR, Xpert amplifies MTB *rpoB’*s RIF resistance-determining region (RRDR). There are five molecular beacons specific for *rpoB* RRDR that detect the presence of MTB and mutations that cause approximately 95% of RIF-R (Chakravorty et al. 2017). Molecular beacons, classified as nucleic acid probes, are deployed to ascertain and report the presence or absence of normal, RIF-sensitive, and “wild-type” sequences within the *rpoB* gene of TB. These beacons emit light in five distinct colors, with each color corresponding to a distinct nucleic acid sequence within the amplified *rpoB* gene (Kohli et al. 2021). Furthermore, the Xpert MTB/RIF test has demonstrated resilience against contamination by MTBDRplus amplicons, rendering it a safe option for laboratory use (Banada et al. 2010; Blakemore et al. 2011). Five generations of cartridges have been introduced: G1, G2, G3, G4, and Xpert Ultra (Ultra), since the inception of the Xpert. Both the Xpert and Ultra versions share a similar approach to sample preparation and cartridge usage (Kay et al. 2020). However, Ultra stands out from the previous Xpert iterations in several technical aspects. It employs two distinct multi-copy amplification targets (IS*6110* and IS*1081*) to enhance the detection of MTB (Kolia-Diafouka et al. 2019). Additionally, Ultra employs a melting temperature-based analysis (MTA) instead of RT-PCR to improve the identification of resistance to RIF (Chakravorty et al. 2017).

### Anyplex MTB/MDR

MTB has a remarkable ability to rapidly develop resistance to drugs such as INH and RIF. As a result, we are witnessing a growing number of patients with TB resistant to RIF and MDR-TB. This surge can be attributed to improper and inadequate patient treatment (Organization 2018). An alternative to the Xpert MTB/RIF, the Anyplex™ II MTB/MDR, offers swift detection of both INH and RIF-resistant MTB (Mpanyane 2015). However, it’s important to note that the WHO has not yet approved the use of the multiplex RT-PCR for identifying DR strains (Organization 2013). A method known as the multiplex RT-PCR, specifically the Anyplex™ II MTB/MDR, has been introduced to indirectly identify the presence of MTBC (Sawatpanich et al. 2022). This particular kit has been applied effectively for the early detection of MTB, particularly in cases involving AFB-positive smear samples (Mpanyane 2015; Sawatpanich et al. 2022).

The Anyplex™ II MTB/MDR is designed for the detection of MTB using dual priming oligonucleotide (DPO™) technology. This technology comprises two priming segments: terminal separator 5 and terminal determinant 3, connected by polydeoxyinosine to create a distinctive “bubble-like structure” (Si et al. 2021). This segment ensures a clear demarcation without actively participating in the priming process, thereby enhancing the sensitivity and specificity of the reaction. Tagging-oligonucleotide cleavage and extension (TOCE${ }^{\text{T}\text{M}}$) is another technology that entails targeting a gene (Huh et al. 2014), allowing for the simultaneous detection of 25 mutations in *katG, inhA*, and *rpoB* genes associated with MDR-TB (Chumpa et al. 2020). However, it’s worth noting that one limitation of this molecular diagnosis method is its inability to identify all drug-resistant genes (Chumpa et al. 2020). The new Anyplex™ II MTB/MDR was developed by Seegene Co. Technologies in South Korea (Igarashi et al. 2017; Nguyen et al. 2019).

### Genedrive MTB/RIF

The Genedrive MTB/RIF offers a detection of TB RIF-resistance which has a combination of paper-based DNA extraction, asymmetric RT-PCR, and a proprietary hybridization probe technology (Highlighter Probes). Laboratories find this method suitable when they have up to eight samples per day (Castan et al. 2014; Nguyen et al. 2019). To illustrate its method, DNA is extracted from bacteria with composite paper based on chemicals. Next, asymmetric RT-PCR is used for two regions of DNA: a short repetitive region which is the REP13E12 family and the 81 bp hotspot region of *rpoB*. Mutation regions of *rpoB* at codons 516, 526, and 531 are detected by the Highlighter probes which have 72.3% sensitivity in detection. When it comes to diagnosis of MTB, between Genedrive and Xpert, Genedrive is better due to 100% sputum samples, the capability of detection as low as five genome copies, the rapid and user-friendly system, and the low price of Genedrive. Moreover, this method is usable for TB point-of-care sites and many AFB smear microscopy centers for screening because of its features of a 12V DC stable power supply, capacity to function without air conditioning, and low-power thermal cycling which its weight is 560 gr and portable (Niemz and Boyle 2012; Organization 2013).

### FluoroType MTB

A swift and precise diagnosis of resistance in the MTB complex (MTBC) is crucial for promptly commencing appropriate diagnosis and treatment. An innovative molecular diagnostic tool, known as the FluoroType MTB, has been introduced to identify resistance to RIF and INH. FluoroType MTB is a molecular method for TB diagnosis (Hillemann et al. 2018). FluoroType MTB involves DNA amplification and detection through PCR within a closed system, with automatic analysis. DNA extraction can be executed either manually or with the assistance of an automated DNA extraction system (GenoXtract) (Hofmann-Thiel and Hoffmann 2014). FluoroType employs an asymmetric PCR approach, utilizing a set of light-on/off probes with a specific device (FluoroCycler® XT) (de Vos et al. 2018). Here’s how it operates: primers amplify distinct amplicons for *inhA*, *rpoB*, and *katG* genes, along with control (de Vos et al. 2018; Haasis et al. 2018). Single-stranded (SS) nucleic acids are identified at the endpoint through melt curve analysis of hybridized sets of on/off probes (de Vos et al. 2018; Hillemann et al. 2018). The cumulative signals from all probes featuring a temperature-dependent fluorescent signature in the same color are interpreted automatically by Fluoro-Software to distinguish each fluorescent signature.

The FluoroType MTBDR displays high sensitivity in identifying RIF resistance in culture isolates of the MTBC but exhibits lower sensitivity in detecting INH resistance. The test is enclosed in a tube, thereby preventing the dispersion of amplicons and eliminating the risk of cross-laboratory contamination. Overall, the advantages of this method encompass reduced processing time, quicker results, diminished risk of acid nucleic contamination, and automatic interpretation with the option for data (Haasis et al. 2018; Hillemann et al. 2018). The FluoroType, when coupled with an automated DNA extraction and PCR setup system, have the potential to enhance the operational efficiency of laboratories in diagnosing drug susceptibility, showing potential for implementation in a molecular drug susceptibility model (Dippenaar et al. 2021).

### TB-CRISPR

Clustered Regularly Interspersed Short Palindromic Repeats (CRISPR) we know as a newly emerged method for gene-editing is the immune mechanism of archaea and bacteria in dealing with bacteriophages (Qian et al. 2022). CRISPR-associated (Cas) proteins combined with CRISPR RNA (crRNA) and trans-acting crRNA (tracRNA) [form the guide RNA (gRNA)] have a functional role in the cleavage viral sequence in bacteria and archaea (Mali et al. 2013). Cas enzymes will be active after attachment of the gRNA molecule. In next step, activated Cas nucleases cleavage the foreign sequence in the host. This fantastic collaboration is usable for detection of nucleic acid for diagnosis of diseases and gives bright future in the diagnosis of different disorders and diseases such as pathogenic diseases for its accuracy and specificity (Wang et al. 2020).

In different studies from 2019, the CRISPR-Cas system was implicated in the detection of specific genes such as virulence genes and nucleotides sequence of MTB (e.g., IS*6110*, IS*1081*, *gyrA, rpoB, katG*, and *inhA*). Cas proteins with neoclassic role have types and classified. Cas13a is a suitable protein for detecting MTB. Although this method and research are not valid by WHO and are in clinical trials, may be the most accurate and practical method in the near future. Ren et. al., worked on improving Cas13a for detection of MTB (Ren et al. 2023). They amplified IS*1081* in 5’ terminal of PCR products T7 is attached. T7 sequence is related to promoter and main factor of these processes. After PCR, T7 was transcripted by specific RNA polymerase and made a ssRNA. This RNA under the guidance of synthesized gRNA (crRNA and tracRNA) binds to Cas13a protein which is a RNase. Finally, this binding is detected by fluorescent probes attached to ssRNA reporters (Fig. 4C). With this new method, we can molecularly diagnose the TB with high-quality diagnosis. If the target sequence is absent, the results will be negative. Other studies with different methodologies are designed and implicated. Most of them amplified DNA by recombinase polymerase amplification (RPA) or LAMP (Huang et al. 2023).

## Utilization, challenges, and the operational process of crafted molecular assays

After countless centuries of contending with MTB, humanity has achieved victory over it. In the primary and pivotal initial phase, our focus should be on pinpointing MTB using purposefully crafted tests. Molecular assays prove highly effective and practical for this specific purpose due to their accessibility and safety. Typically, within molecular assays, we identify MTB by scrutinizing the genes responsible for various characteristics (such as drug resistance, virulence factors, and proteins) or by investigating SNPs (Rabaan et al. 2022). We have delineated a selection of specially designed molecularassays centered around genome identification. Diverse genes play crucial roles in these assays, including *rpoB*, *gyrA*, *inhA*, and *katG*, each serving distinct functions (Seid et al. 2022). Practical methodologies and assays for the detection of MTB have been detailed in Table 1. According to the development of molecular science and its competition with microbial intelligent agents, it is obvious that we will observe to improve the assays for detecting and identifying MTB in the coming years and decades.

**Table 1:** Utilization, challenges, and operational processes of molecular assays that have been created

Assays	Utilization	Challenges	How it works?	References
RT-PCR	Identification of different genes in the simplest way	Low sensitivity	After PCR, detect the amplified genes by connecting a probe and identify them based on fluorescent detection and/or melting curve analysis	(Rao et al. 2016)
Microarray	Identify many genes at the same time with high accuracy	Low sensitivity and diagnosis and needed improvement	In a typical microarray, labeling, and wiping desired nucleic acid fragments with a fluorescent dye during PCR	(Butcher 2004; Nguyen et al. 2019)
PURE-LAMP	Detect MTB quickly and accurately	Increases the economic burden on patients Is not able to identify and diagnose drug-resistant patterns	Three stages: sample preparation, amplification, and detection by UV light from the tube	(Neonakis et al. 2011; Zijenah 2018)
NGS	Sequencing of MTB for mutated resistance genes	High-cost, difficulty	After PCR and cell preparation, the library should select and apply, sequencing by devices, and finally bioinformatical analysis	(Bagratee and Studholme 2024; Beviere et al. 2023)
RFLP analysis	Analysis of IS*6110*	Obsolete due to the rise of inexpensive DNA sequencing technologies	RFLP analysis: this method is one of the oldest methods to detect MTB. In this method, DNA after amplification, is restricted by enzymes and labeling. next, gel electrophoresis is done for the separation of different fragments of DNA. Detection of fragments is done with laser emission	(Caws et al. 2007; Hayward 1995)
WGS	Detect resistance to disinfectants, antimicrobials, and the relationship between isolates and the source of contamination Detect resistance and predict sensitivity and trace transmission of MTB Allows the detection of mutations outside the target area of the assays	WGS alone is not reliable to investigate the full prevalence of the disease	The WGS has various steps, including DNA extraction, sequencing, and data analysis In WGS, extraction and purification of DNA is done first. The DNA is then split into smaller pieces to be sequenced. The results should be analyzed quickly in a clear way to enable the use of WGS data	(Katale et al. 2020; Witney et al. 2016)
Truenat	Resistance detection	Unavailable for everyone, specific reagents, and master mix	RT-PCR done on the designed chip in the device after the DNA extraction by Trueprep kit	(Inbaraj et al. 2023; Ngangue et al. 2022)
Cobas TaqMan MTB	For liquid, disinfected, and concentrated samples of respiratory patients The ability to accurately identify MTB in a suspect test	Cause false negative test results, we can mention the uneven distribution of bacteria in the sample, the presence of enzyme amplification inhibitors, and the small volume of the sample More expensive than the traditional cultural method, but it has a wide contribution to the detection of MTB	Two steps, including sample DNA preparation and RT-PCR The sample is first sterilized and then centrifuged at 60 °C for 45 min and lysed. The mixture is prepared and DNA is added, the tubes are placed in the Cobas TaqMan analyzer for RT-PCR	(Eddabra and Ait Benhassou 2018a; Yang et al. 2011)
E-MTD	The direct tests for the detection of MTB in the sample Ability to detect both smear-positive and smear-negative pulmonary TB,	Including false positive or negative results, and the interpretation of the test results should be based on the clinical picture	The rRNA of the target cell is released and amplified, then the reaction product is labeled. These reactions require constant temperature	(Greco et al. 2006; Smith et al. 1999)
LPA	Helps to detect drug resistance. This qualitative laboratory test for the identification of MTBC and its resistance	Very sensitive and specific for detection of MDR-TB	Is performed by DNA extracted from culture or directly from clinical samples. Genes are amplified using PCR. In the next step, PCR products are biotinylated, and immobilized probes are hybridized. Then the results are determined by colorimetric development	(Desikan et al. 2017; Noor et al. 2015)
Xpert MTB/RIF	It is a relatively new WHO-recommended, nucleic acid amplification assay that detects MTB and RIF resistance at the same time	Has obstacles such as in poor countries, its cost, environmental restrictions (stable and regular electricity, suitable room temperature), and problems in supply and maintenance	Detects MTB complex and mutations associated with RIF resistance in the *rpoB* gene. Results are available with minimum technical time within two hours after the start. The Xpert assay integrates DNA extraction, PCR, and detection into a self-contained test unit	(Zifodya et al. 2019)
Anyplex™ II MTB/MDR	Rapidly detects both INH-resistant and RIF-resistant TB	Has not been approved by WHO Is not identifying all drug-resistant genes	It consists of two priming segments, terminal separator 5 and terminal determinant 3. The two are linked by polydeoxyinosine to form a “bubble-like structure”. This section creates a clearer boundary without participating in the priming process by fixing the end of 5 and the end of 3	(Chumpa et al. 2020; Singh et al. 2019)
Genedrive	Detection of *rpoB* mutation and RIF resistance	Just determine one drug resistance	DNA extraction, asymmetric RT-PCR, and a proprietary hybridization probe technology	(Niemz and Boyle 2012; Shenai et al. 2016)
FluoroType	Available for the detection of resistance to RIF and INH	Less sensitivity for detecting INH resistance	Includes DNA amplification and detection through PCR in a closed system with automatic analysis	(Dippenaar et al. 2021; Zabost et al. 2022)
TB-CRISPR	Detect and identify the target sequences such as IS*6110* and IS*1081*	Difficulty and invalidated by WHO and FDA	The promoter of the target sequence after amplification, is bound to the Cas protein with the guide of gRNA, and signaling RNAs will attach to the complex and emit the fluorescent light	(Zhang et al. 2023)

## Diagnostic molecular assays at a glance

The complexity of MTB and the various challenges associated with its detection mandate diagnostic methods that are not only highly sensitive but also capable of discerning drug resistance patterns and strain diversity. Molecular assays, underpinned by the principles of NAA and detection, have emerged as indispensable tools in the armamentarium against TB. Molecular assays offer several key advantages over traditional methods, making them essential in addressing the gaps and limitations of current TB diagnostics (MacLean et al. 2020). First and foremost, they exhibit remarkable sensitivity, allowing for the detection of low numbers of MTB bacilli, which is especially crucial in cases of paucibacillary and extrapulmonary TB (Boehme et al. 2010). Second, these assays can rapidly provide results, often within hours, facilitating prompt initiation of treatment and reducing the risk of disease transmission. Additionally, molecular assays can simultaneously detect MTB and its drug resistance profiles, enabling tailored therapeutic interventions and containment of MDR-TB (Lawn et al. 2011). Finally, these assays can discriminate between MTB strains, aiding in epidemiological investigations and the identification of emerging strains of concern (Walker et al. 2015).

The development of molecular assays for MTB detection has been a dynamic process, marked by continuous innovation and refinement. The pivotal breakthrough was the advent of PCR-based methods in the 1980s, which allowed for the amplification of specific MTB DNA sequences. Subsequent adaptations, including RT-PCR and nucleic acid hybridization, further improved sensitivity and reduced the risk of contamination (Liu 2011). The landscape of MTB molecular diagnostics witnessed a transformative shift with the introduction of the Xpert MTB/RIF assay. This fully automated platform combines RT-PCR with molecular beacon technology, enabling rapid detection of MTB and simultaneous determination of RIF resistance, a surrogate marker for MDR-TB (Boehme et al. 2010). The GeneXpert system has revolutionized TB diagnostics in resource-limited settings, where its user-friendliness and minimal infrastructure requirements have made it an invaluable tool for TB control programs (Helb et al. 2010).

While PCR-based assays have been instrumental in TB diagnosis, recent advancements have expanded the molecular toolbox for TB diagnosis. Isothermal amplification techniques, such as LAMP, offer several advantages over PCR, including simplified instrumentation requirements, faster amplification, and improved tolerance to inhibitory substances in clinical specimens (Notomi et al. 2000; Organization 2022). LAMP-based assays targeting MTB have shown promise in enhancing accessibility to TB diagnostics, particularly in resource-constrained settings (Mitarai et al. 2011). Moreover, the era of NGS has ushered in a new dimension of MTB molecular analysis. NGS not only enables the comprehensive characterization of MTB genomes but also facilitates the identification of genetic determinants associated with drug resistance and virulence (Jagadeesan et al. 2019). The application of NGS in TB diagnostics holds immense potential for unraveling the intricacies of MTB biology and epidemiology, ultimately contributing to more effective disease control (Dookie et al. 2022b).

## Future perspective for TB and its molecular diagnosis

The development of molecular diagnostics offers promise for the future management of TB. A cost-effective, accurate, and rapid diagnostic tool is urgently needed to improve disease diagnosis and treatment initiation with the global burden of TB persisting. The advent of novel technologies (e.g., NGS, and CRISPR-based detection) has revolutionized the diagnosis of TB, enabling highly sensitive and specific detection of MTB directly from clinical samples. Aside from expediting diagnosis, these assays facilitate identifying DR strains, which guides the development of individualized treatment plans. Furthermore, the integration of AI algorithms with molecular diagnostics can further refine TB diagnosis and prognosis by enhancing the interpretation of complex genetic data. TB management can be transformed through the adoption of these technologies, which can lead to earlier detection, improved treatment outcomes, and ultimately, a reduction in TB-related mortality and morbidity.

As we gain a deeper understanding of the molecular mechanisms underlying TB pathogenesis, we can develop targeted therapies as well as vaccines to combat the disease. Precision medicine approaches tailored to individual patients can be developed by identifying biomarkers associated with TB progression and treatment response. Moreover, the advent of new vaccine platforms, including viral vectors and mRNA vaccines, offers hope for the development of more effective TB vaccines capable of conferring durable protection from infection and disease progression. Immunogenetics and systems biology advances are making it possible to design vaccines that elicit robust and long-lasting immune responses, potentially overcoming the limitations of current Bacillus Calmette-Guérin (BCG) vaccination strategies. Furthermore, the integration of omics technologies, including genomics, transcriptomics, proteomics, and metabolomics, is providing unprecedented insights into host-pathogen interactions and immune responses to TB infection, paving the way for the development of novel therapeutics and vaccines. By harnessing these multidisciplinary approaches, the future holds promise for a comprehensive strategy to combat TB, from early diagnosis to targeted treatment and prevention.

## Abbreviations

AFB: Acid-fast bacilli
AI: Artificial intelligence
AMK: Amikacin
Cas: CRISPR-associated
cDNA: Complementary DNA
CRISPR: Clustered regularly interspersed short palindromic repeats
CrRNA: CRISPR RNA
COVID-19: Coronavirus disease 2019
Cy: Cyanine
DdRp: DNA-dependent RNA polymerase
DDW: Double distillation water
DPO: Dual priming oligonucleotide
DST: Drug susceptibility test
E-MTD: Gen-probe enhanced MTB Direct
FDA: Food and Drug Administration
gRNA: Guide RNA
HCR: Hybridization chain reaction
HIV: Human immunodeficiency virus
HTST: High-throughput sequencing technologies
INH: Isoniazid
LED-FM: Light-emitting diode-fluorescent microscopy
LJ: Löwenstein–Jensen
LPA: Line probe assay
LZD: Linezolid
MDR: Multi-drug resistance
MIRU-VNTR: Mycobacterial interspersed repetitive-unit–variable-number tandem-repeat
MTA: Melting temperature-based analysis
MTB: *Mycobacterium tuberculosis*
MTBC: MTB complex
NAATs: Nucleic acid amplification techniques
NCBI: National Center for Biotechnology Information
NGS: Next-generation sequencing
ORF: Open reading frame
PCR: Polymerase chain reaction
PZA: Pyrazinamide
PURE-LAMP: PURE-Loop-mediated isothermal amplification
PURE: Procedure for ultra-rapid extraction
RFLP: Restriction fragment length polymorphism
RRDR: Rifampin resistance-determining region
RH: Reverse hybridization
RIF: Rifampin
RPA: Recombinase polymerase amplification
RT-PCR: Real-time PCR
SNP: Single nucleotide polymorphism
SSM: Spot-spot-morning
SMS: Spot-morning-spot
SNVs: Single nucleotide variations
STR: Streptomycin
TBDR: Tuberculosis drug-resistance
TB: Tuberculosis
TOCE: Tagging-oligonucleotide cleavage and extension
TracRNA: Trans-acting crRNA
TGS-TB: Total genotyping solution for TB
TGS: Total genotyping solution
UV: Ultraviolet
VNTR: Variable-number tandem repeat
WHO: World Health Organization
WGS-TB: Whole-genome sequencing for TB
